# Posture Deficits and Recovery After Unilateral Vestibular Loss: Early Rehabilitation and Degree of Hypofunction Matter

**DOI:** 10.3389/fnhum.2021.776970

**Published:** 2022-02-04

**Authors:** Michel Lacour, Laurent Tardivet, Alain Thiry

**Affiliations:** ^1^Neurosciences Department, Aix-Marseille University/CNRS, Marseille, France; ^2^21 Impasse des Vertus, Fuveau, France; ^3^Otorhinolaryngology Department, CHU Nice, Nice, France; ^4^Private Practitioner, Nice, France

**Keywords:** unilateral vestibular loss patients, posture and balance control, vestibular rehabilitation, early vs. late rehab, degree of vestibular hypofunction

## Abstract

Postural instability and balance impairment are disabling symptoms in patients with acute unilateral peripheral vestibular hypofunction (UVH). Vestibular rehabilitation (VR) is known to improve the vestibular compensation process, but (1) its effect on posture recovery remains poorly understood, (2) little is known about when VR must be done, and (3) whether the degree of vestibular loss matters is uncertain. We analyzed posture control under static (stable support) and dynamic (unstable support) postural tasks performed in different visual conditions [eye open (EO); eyes closed (EC); and optokinetic stimulation] using dynamic posturography. Non-linear analyses of the postural performance (wavelet transform, diffusion analysis, and fractal analysis) were performed in two groups of patients with UVH subjected to the same VR program based on the unidirectional rotation paradigm and performed either early (first 2 weeks) or later (fifth to the sixth week) after vertigo attack. Distribution of the angular horizontal vestibulo-ocular reflex (aVOR) gain values recorded on the hypofunction side before rehabilitation differentiated two distinct sub-groups (cluster analysis) with aVOR gains below or above 0.20. The postural performance of the four sub-groups of patients with UVH (early rehabilitation with aVOR gain <0.20: *n* = 25 or gain >0.20: *n* = 19; late rehabilitation with aVOR gain <0.20: *n* = 15 or gain >0.20: *n* = 10) tested before VR showed significantly altered postural parameters compared with healthy controls. Greater instability, higher energy to control posture, larger sway without feedback corrections, and lower time of automatic control of posture were observed in static conditions. The four sub-groups recovered near-normal postural performance after VR in the EO and EC conditions, but still exhibited altered postural performance with optokinetic stimulation. In dynamic posturography conditions and before VR, the percentage of patients able to perform the postural tasks with EC and optokinetic stimulation was significantly lower in the two sub-groups with aVOR gain <0.20. After VR, the improvement of the postural parameters depended on the stage of rehabilitation and the degree of vestibular hypofunction. The best balance function recovery was found in the sub-group with early VR and pre-rehabilitation aVOR gain above 0.20, the worst in the sub-group with late rehabilitation and aVOR gain below 0.20. These differences were seen when the vestibular input remains the main sensory cue to control balance, that is, on unstable support without vision or altered visual motion cues. These findings extend to dynamic balance recovery the crucial roles of early rehabilitation and degree of vestibular hypofunction which we have already highlighted for vestibulo-ocular reflex recovery.

## Introduction

Body stabilization and orientation in space are the main goals of the postural control system (Massion, 1994). Allocentric (vision), egocentric (somatosensory), and geocentric (vestibular) reference frames contribute to posture control through multisensory integration in the subcortical and cortical nervous structures. In everyday life, quiet standing in healthy subjects is automatically regulated. Postural adjustments, if necessary, are made by visual and somatosensory feedback mechanisms that predominate over vestibular input for body orientation and stabilization (Fitzpatrick and McCloskey, 1994). The tonic activation of anti-gravity muscles through the lateral and medial vestibulo-spinal pathways is the main vestibular contribution to quiet standing (Lacour and Borel, 1993; Curthoys et al., 2017). Whereas genetic models of posture control rely on such sensory integration and reflexes, cognitive models focus on the internal representation of body position in space (Massion, 1994; Mergner and Rosemeier, 1998). Internal models are used to forward motor commands taking into account the environmental constraints. The strategy used for balance control depends on both environmental context and pathological conditions (Borel et al., 2008). In more challenging postural tasks on unstable supports with eyes closed (EC) or with moving visual surrounds, vestibular inputs become more crucial to keep balance. Dynamic posturography findings in healthy subjects and vestibular loss patients clearly showed changes in sensory cues weighting under sway referenced visual or somatosensory contexts (Nashner et al., 1982; Black et al., 1983).

Animal models of vestibular loss have identified the vestibular contribution to static and dynamic posture control (Lacour et al., 1976; Xerri and Lacour, 1980; Dieringer, 1995). After an acute peripheral unilateral vestibular loss, increased support surface, head and trunk tilt, falls and deviation of the locomotor trajectory to the lesion side, and loss of dynamic balance function were observed. Similar static and dynamic postural deficits have been described in patients with unilateral vestibular loss (Black et al., 1988, 1989) who showed ipsilateral roll and frontal head tilt (Borel et al., 2002), abnormal body alignment (Horak and Shupert, 1994), increased body sway with eyes open (EO) and EC (Black et al., 1983; Allum et al., 1988; Fetter et al., 1991; Lacour et al., 1997), impaired locomotor pattern and ipsilateral falls (Borel et al., 2004), and poor postural performances in dynamic posturography when vision or somatosensory inputs were sway referenced (Nashner et al., 1982; Black et al., 1983; Horak and Shupert, 1994; Borel et al., 2002). Postural deficits recovery in easy postural tasks was generally seen over weeks and months as the result of sensory substitution mechanisms based on visual and somatosensory inputs. Recovery of dynamic balance function took more time, however, and showed incomplete compensation in more challenging postural conditions (Lacour et al., 1997, 2020a; Curthoys and Halmagyi, 1999; Lacour, 2006; Horak, 2010).

Vestibular rehabilitation (VR) therapy is effective for improving balance, dizziness, and quality of life in patients with vestibular loss (Hillier and McDonnell, 2011, 2016; Whitney et al., 2015). Many clinical investigations have demonstrated that VR is safe and effective, accelerates the recovery process, optimizes the final level of vestibular compensation, and restores a good quality of life (Lacour and Bernard-Demanze, 2014). However, when posture rehabilitation must be done remains to be clarified; the optimum stage for performing VR has been a matter of debate, although no clear answers have emerged until recently. Data recorded in patients with unilateral peripheral vestibular hypofunction (UVH) rehabilitated with gaze stability exercises (Lacour et al., 2019) or the unidirectional rotation paradigm (Lacour et al., 2020b) reported better angular horizontal vestibulo-ocular reflex (aVOR) gain recovery with early rehabilitation (the first 2 weeks) compared with later rehabilitation. The degree of vestibular loss was a second crucial parameter influencing aVOR recovery: patients with pre-rehabilitation aVOR gain above 0.20 fully recovered dynamic canal function compared with patients with lower gain who used covert saccades to stabilize gaze (Lacour et al., 2021). Do early rehabilitation and degree of vestibular loss also matter for balance recovery in patients with UVH? In other words, is there a critical period for rehabilitation of posture and balance, and is the degree of vestibular hypofunction on the weaker side another important parameter to recover balance function? On the basis of our recent findings on dynamic canal function recovery (Lacour et al., 2021), we tested the hypothesis that patients with early rehabilitation and higher pre-rehabilitation aVOR gain would recover dynamic equilibrium better than those with late rehabilitation and lower aVOR gain.

This retrospective study on 69 patients with UVH was aimed at testing this hypothesis. The four subgroups of patients were subjected to the same VR therapy program (unidirectional rotation paradigm, training sessions twice a week for 4 weeks). Posturography recordings were made just before starting VR and at the end of the last VR session. Static and dynamic posturography data were analyzed separately.

## Materials and Methods

### Participants

The 69 patients with UVH (vestibular neuritis) were diagnosed based on patients’ history and clinical examination. The criteria defined by the Barany Society (Strupp and Magnusson, 2015) were used for patient inclusion, that is, acute onset of spinning vertigo, postural imbalance, nausea, spontaneous horizontal rotatory nystagmus, and positive head impulse test (HIT). The patients underwent passive HIT to quantify the horizontal aVOR gain using the VHIT Ulmer recording device (Synapsis, Marseille, France) and to evaluate the degree of vestibular loss before VR. All patients showed pathological aVOR responses on the hypofunction side, with aVOR gains below 0.65, and normal responses (gain >0.80) on the healthy side (Table 1). Among the 69 patients, 55 had pathological HIT responses to horizontal and vertical anterior canal tests on the hypofunction side, attesting impairment of the superior branch of the vestibular nerve. The remaining 14 patients had pathological HIT tests for the horizontal, vertical anterior, and posterior canal tests, showing impairment of both the superior and inferior nerve branches. The vestibular syndrome is similar in patients with complete impairment and patients with the superior branch only, and their handicap scores as well, all patients have been included in the present study. The caloric test was not systematically performed because of the discomfort caused to patients, particularly when performed in the acute stage, but when done, the response was lacking on the lesion side. The vestibular evoked myogenic potentials were not done due to the lack of the necessary equipment. Central vestibular or ocular motor dysfunctions, positional vertigo, and drug treatments constituted the exclusion criteria. After inclusion, the patients were advised not to take anti-vertigo drugs. Written informed consent to participate had been obtained for each patient and the investigation was performed according to the Helsinki Declaration and ethics local committee (CCPPRB) approval.

**TABLE 1:** Main characteristics of the four sub-groups of patients with UVH.

*N* = 69	Early rehabilitation		Late rehabilitation	
	aVOR gain <0.20 (*n* = 25)	aVOR gain >0.20 (*n* = 19)	aVOR gain <0.20 (*n* = 15)	aVOR gain >0.20 (*n* = 10)
Mean aVOR gain (±SD)	0.08 ± 0.05***	0.44 ± 0.13	0.08 ± 0.04***	0.35 ± 0.14
Time from onset (days)	8.0 ± 4.6	9.0 ± 3.4	32.4 ± 8.9***	32.5 ± 6.9***
SPEV (°/s)	8.97 ± 5.58	6.15 ± 2.66	3.51 ± 2.50***	3.95 ± 2.86***
Age (years)	62.4 ± 14.2	56.5 ± 15.0	63.1 ± 14.4	66.4 ± 15.7
DHI score	63.9 ± 15.4	54.8 ± 17.6	56.7 ± 18.7	52.7 ± 22.9

The whole population of UVH patients (N = 69) was divided into four sub-groups with early rehabilitation and pre-rehabilitation aVOR gain below 0.20 (n = 25) or above 0.20 (n = 19), and with late rehabilitation and pre-rehabilitation aVOR gain below (n = 15) or above (n = 10) 0.20 (see section “Materials and Methods”: cluster analysis). The table shows the mean (±SD) of aVOR gain measured at the inclusion visit, the time delay between symptoms onset and beginning of vestibular rehabilitation (days), slow phase eye velocity (SPEV) of the spontaneous nystagmus recorded in darkness (°/s), and age (years) recorded in the four subgroups of patients with UVH. The dizziness handicap inventory (DHI) score evaluated at the inclusion visit shows that all patients were in the range of moderate handicap. Significant differences depending on the degree of vestibular hypofunction (aVOR gain >or <0.20) and on early vs. late rehabilitation are indicated by asterisks. ***p < 0.001.

All patients were in the range of moderate handicap, with dizziness handicap inventory (DHI) scores ranging from 40 to 60 points, and in the same age range. Table 1 summarizes the main characteristics of the four subgroups of patients. The data collected in healthy age-matched subjects (*N* = 225) were used for comparison with the patients’ postural performance recorded in static posturography conditions.

### Measurement of the Horizontal Angular Vestibulo-Ocular Reflex Gain

The HIT was used to evaluate the horizontal aVOR gain on both intact and hypofunction sides just before the very first VR session and at the end of the last VR session. The patients were tested while seated with the head tilted down by 30° to put the horizontal semicircular canal in the horizontal plane. Head rotations to the healthy and weaker sides were done passively with 10° peak amplitude, 200°/s peak velocity, and around 2,000°/s^2^ peak acceleration. Head thrust tests were performed randomly to elicit unpredictable HITs concerning the timing and direction of head movement. The recording of the vertical aVORs was done by turning the patient’s head 45° to the right (LARP) and then to the left (RALP). The gain values of the aVOR were calculated by the ratio of peak eye velocity/peak head velocity. A mean gain was obtained from several HITs performed in the same semicircular canal plane. The VHIT Ulmer software (Synapsys) measures the gain of the vestibulo-ocular reflex with an accuracy of 0.1° for the angular position of the eye. Many more than 5 trials were generally done due to blinks or the absence of perfect focus on the target by the patients. An average gain value was calculated before VR and after VR from five correctly performed tests on both sides.

### Static and Dynamic Posturography

#### Experimental Setup

Posturography tests have been fully described previously (Lacour et al., 2020a). Briefly, the Center of foot Pressure (CoP) displacements were recorded with a force platform (Multitest Equilibre, Framiral, Grasse, France: sampling frequency: 50 Hz) during six consecutive sequences of 30 s each separated by 15–30 s rest periods. The patients were instructed to stand as quietly as possible without head/body voluntary motion. The first three sessions were performed on the fixed support (stable support, static posturography) with EO, EC, and in front of a moving random visual pattern provided by an optokinetic device (Opto: Framiral, Grasse, France). The last three sessions were performed in the same visual conditions (EO, EC, Opto) on the three-dimensional (3D) free-moving platform (unstable support, dynamic posturography). Randomization of the trials (stable vs. unstable platform, EO, EC, and Opto) has not been done as it is preferable to start with easy postural tasks in patients with UVH tested at the acute stage of their vestibular deficit and to progressively increase postural task difficulty. Body sway was evaluated in each visual condition by computing the CoP displacement over time in the anteroposterior and mediolateral directions.

#### Data Processing

Non-linear analyses of CoP displacements were used to provide an accurate evaluation of posture and balance control. The wavelet transform, the stabilogram diffusion analysis, and the fractional Brownian-motion analysis were applied to the stabilograms (PosturoPro software, Framiral, Grasse, France).

**FIGURE 1:**
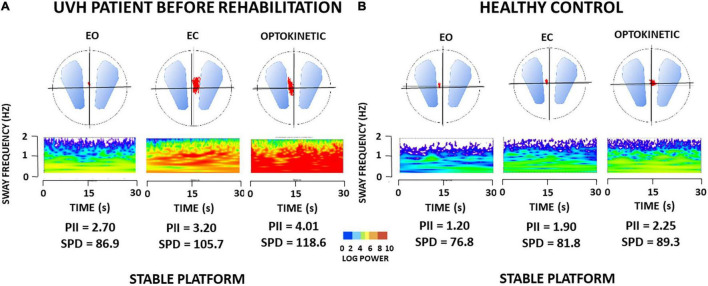
**(A,B)** Wavelet transform of one representative patient with unilateral vestibular hypofunction (UVH) examined before rehabilitation, and of one healthy subject in static posturography conditions. Wavelet analysis performed on the antero-posterior center of foot pressure (CoP) displacements (red area in the circles) provides three-dimensional (3D) charts of body sway with time (s) on the abscissae, body sway frequency content (Hz) on the ordinates, and spectral power density (decimal Log Power) as color code. **(A)** 3D maps of the patient with UVH [early rehabilitation subgroup with an angular horizontal vestibulo-ocular reflex (aVOR) gain above 0.20] tested before rehabilitation on a stable platform with eyes open (EO), eyes closed (EC), and with optokinetic stimulation (Optokinetic), and **(B)** 3D maps of sex- and age-matched healthy subject. The figure shows higher body sway frequencies and much more energy spent to control posture in the patient compared to the healthy subject, whatever the visual condition. Values of postural instability index (PII) and spectral power density (SPD) derived from the wavelet analysis are reported below each recording.

Methodologically, the wavelet analysis is appropriate to study non-stationary signals such as CoP displacements, without presenting the limitations of the Fast-Fourier Transform. It provides a time-frequency chart of body sway in the 3D space, giving access to the changes in body sway frequency components with time (see 32–33 for details). The spectral power density (SPD) was expressed as a decimal logarithm scale reported on the 3D map by a color code (see Figure 1). It is a good estimate of the energy cost required to maintain a stable postural performance. SPD was calculated in the frequency domain of the visual system (0.05–0.50 Hz). The postural instability index (PII) was elaborated from the SPD contained in the stabilogram and the cancellation time, that is, the time during which spectral power of the different body sway frequencies tend to be close to zero by closed-loop control mechanisms. The PII is computed as a global score quantifying posture stability. The higher the PII, the higher the instability, and the higher the SPD, the higher the energy spent to control posture.

The stabilogram diffusion analysis (Collins and De Luca, 1993) computes the square of the CoP displacements between all pairs of points separated by a time interval Δt, then averaged over the number of Δt in the recording session, and repeated for increasing values of Δt. The planar stabilogram-diffusion plot defines a critical point at which the spatio-temporal coordinates approximate the region over which posture control switches from open-loop to closed-loop control mechanisms. The amplitude of the critical point (CP amp, in mm^2^) estimates when feedback mechanisms intervene to avoid falls. The higher the CP amplitude, the higher the risk of falls.

The fractal analysis is based on fundamental concepts and principles from statistical mechanics (Einstein, 1905). It is aimed at determining if two consecutive points in the stabilogram are causally related (CoP moving forward because of previous backward displacement: feedback correction; closed-loop control mechanism), or if the sampling points in the CoP trajectories are not linked by a causal relationship (Hausdorff points: random CoP trajectory, stochastic process: open-loop control mechanism). The total number of Hausdorff points in each stabilogram was evaluated and the mean Hausdorff frequency (HF) was calculated. The HF parameter provides another estimate of posture stability. It allows approximating the mean time interval (*t* = 1/HF) during which patients remain stable without doing any postural correction. The higher the HF and the more frequently the postural system operates in an open-loop, automatic control, the higher the posture stability.

### Vestibular Rehabilitation: The Unidirectional Rotation Paradigm

The rotatory chair protocol was used as a VR therapy protocol able to rebalance the vestibular system (Lacour et al., 2019, 2020b). Briefly, the patients with UVH sitting in the rotatory chair (Framiral, Grasse, France) were subjected to whole-body rotations toward the hypofunction side. The rotations consisted of sudden high-velocity rotation of the chair (200–250°/s; acceleration: 1,000°/s^2^) during three full 360° turns or more, followed by a sudden stopping of the chair at the end of the last lap. The patients were instructed to keep their EC during the whole rotation and the head tilted by 30° down to put the horizontal canal plane close to the horizontal. Five to 10 trials were successively done during the same session, depending on the patient’s tolerance of the protocol, with a maximum duration that did not exceed 30 min. Neurovegetative symptoms could be observed during whole-body rotation (pallor and sweating in around 20% of the patients), but stopping rehabilitation for the day was rare (5% of the patients). When necessary to stop the training session, the physiotherapist started the next session with a reduced rotation speed. The frequency of chair rotation was around 0.005 Hz, including a resting period of 120–150 s between each chair rotation. This VR paradigm was found to reduce the contralateral intact horizontal VOR response in animal models of unilateral vestibular loss (Ushio et al., 2011), and to increase the VOR gain on the hypofunction side in unilateral vestibular patients (Sadeghi et al., 2018). The four subgroups of patients with UVH in the present study were subjected to the same training protocol, including an equal number of training sessions. The rehabilitation sessions were performed twice a week for 4 weeks after the inclusion of the patients, with the first rehabilitation session done just after the inclusion visit.

### Data Analysis

Distribution of the aVOR gain values recorded in the early (*N* = 44) and late (*N* = 25) rehabilitation groups have been constructed by pooling the individual values per 0.05 class intervals. A bimodal distribution pattern was observed in both groups, indicating that these two populations were not homogeneous. The Shapiro–Wilk test confirmed that Gaussian distributions were not found (*W* = 0.886, *p* = 0.0004; *W* = 0.795, *p* = 0.0014 for the early and late groups, respectively). To determine whether there was a bimodal distribution or a skewed distribution, the data were subjected to cluster analysis. This procedure was used to provide independent, statistical criteria, and was performed with the *K*-means splitting method (Systat software: version 5.0, San Jose, CA, United States). It provides the best-partitioned clusters based on a statistical analysis in which the groups are not known in advance. The cluster analysis clearly split the early and late rehabilitation groups of patients with UVH into two well-identified and significantly different clusters (*p* < 0.0001), defining therefore two different sub-populations in each group of patients with UVH, with aVOR gain values either below 0.20 or above 0.20.

To evaluate the vestibular loss-induced deficits observed in static posturography conditions, the pre-rehabilitation postural performance recorded in the four sub-groups of patients with UVH was compared with the data collected in healthy age-matched subjects. Such comparison could not be done in dynamic posturography conditions, most of the patients being unable to keep balance on unstable support without vision or with moving visual motion cues. Posture recovery was analyzed using a four-way ANOVA followed by *post hoc* analysis with the Tukey test (Stateview II software, UCS, CA, United States). ANOVAs were done with sub-groups (aVOR gain below vs. above 0.20, early vs. late rehabilitation) and the four parameters describing body sway [PII; SPD; critical amplitude (CA); HF] as between-patients factors, and pre-rehabilitation vs. post-rehabilitation data as the within-patients factors.

In contrast to healthy subjects, many patients examined before rehabilitation failed to keep balance in the most challenging dynamic posturography conditions (EC and optokinetic stimulation on unstable support). The patients were not equipped with a safety harness, but they could cling to the security bar surrounding the platform. Grasping the bar with the hands was considered a fall. The percentage of fallers was evaluated before and after rehabilitation as a functional parameter to assess the effects of both early vs. late VR and the degree of vestibular loss. Given the small size of each subgroup of patients with UVH tested in dynamic posturography conditions, and the values that did not always follow a normal Gaussian pattern, the statistical analysis was performed with non-parametric tests. Indeed, the distribution of the aVOR gain values showed asymmetrical shape histograms with positive skewness in the two sub-groups with aVOR gains below 0.20. It was more symmetrical in the sub-groups with aVOR gains above 0.20. The four sub-groups were compared with the Kruskal–Wallis test. Results were considered significant for *p* < 0.05.

## Results

### Angular VOR Gain Distribution on the Hypofunction Side Before Rehabilitation

All patients with UVH showed normal horizontal aVOR gain values (>0.80; HIT) on their intact side.

Table 1 summarizes the mean (±SD) aVOR gain values recorded in the four well-identified sub-groups on the hypofunction side. In the early rehabilitation patients, the bimodal distribution pattern showed a first sub-group (*n* = 25) with aVOR gain values of 0.08 ± 0.05 and a second one (*n* = 19) with aVOR gains of 0.44 ± 0.13 (*p* < 0.001). Similar findings were observed in the late rehabilitation patients with gain values of 0.08 ± 0.04 (*n* = 15) and 0.35 ± 0.14 (*n* = 10) for the two sub-groups, respectively (*p* < 0.001). The two one-sided tests showed the equivalence of the means for both sub-groups with aVOR gain below and above 0.20 (*p* < 0.0001). Mean age and DHI scores were similar in the four sub-groups, all patients with UVH being in the same range of moderate handicap. The time delay between symptoms onset and beginning of rehabilitation was significantly different between the early vs. late sub-groups (∼8–9 vs. ∼32 days). And the slow phase eye velocity of the spontaneous nystagmus also differed significantly between these sub-groups as a result of spontaneous compensation occurring over time (*p* < 0.001; see Table 1).

### Posture Control in Static Posturography Conditions

#### Before Rehabilitation

As a rule, all patients with UVH were able to keep balance in the postural tasks performed on a stable support. Figure 1A shows the wavelet transform applied to the stabilograms of one representative patient with UVH tested before VR in the three static posturography conditions with EO, EC, and with optokinetic stimulation (Optokinetic). Raw wavelet plots are shown as 3D maps with body sway frequency and power density as a color code in the frequency domain, according to recording time. The 3D maps of one representative age-matched healthy control are illustrated in Figure 1B. Comparison of the PII and SPD parameters derived from the wavelet analysis shows increased body instability and higher energy to keep the balance for the patient compared to the healthy control.

The ANOVAs performed on each of the posturography parameters pointed to significant differences between the patients and the healthy subjects. The values were [*F*_(4,292)_ = 76.24; *p* < 0.001] for PII, [*F*_(4,292)_ = 69.41; *p* < 0.001] for SPD, [*F*_(4,292)_ = 48.45; *p* < 0.001] for the amplitude of the critical point, and [*F*_(4,292)_ = 39.50; *p* < 0.001] for HF.

Table 2 (left part) provides the mean PII scores derived from the wavelet transform. The PII scores recorded in the patients under static posturography with (EO), without (EC), and altered (Optokinetic) vision were significantly increased compared to the controls (*p* < 0.001 for the three visual conditions). The biggest increase was found with optokinetic stimulation. Compared with the healthy controls, the wavelet analysis also indicated that patients with UVH spent more energy to control posture (*p* < 0.001). The diffusion analysis showed that they shifted to close-loop control mechanisms for higher CoP displacements (*p* < 0.01), and the fractal analysis indicated that they were unstable over longer periods (*p* < 0.01) (not illustrated). Taken together, all postural parameters elaborated with different non-linear analyses pointed to the strong impairment of quiet standing in the three visual conditions. ANOVA did not show significant differences between the four sub-groups [*F*_(3,67)_ = 62.3; *p* = 0.72] whatever the data processing used. This indicates that the patient postural performance tested in static conditions depended neither on the degree of vestibular loss (aVOR gains below or above 0.20) nor on the time delay between symptoms onset and beginning of VR (early vs. late rehabilitation).

**TABLE 2:** Effect of vestibular rehabilitation on patients’ posture stability in static posturography conditions.

	Postural instability index			Postural instability index		
	Before rehabilitation			After rehabilitation		
Groups	Stable EO	Stable EC	Stable Opto	Stable EO	Stable EC	Stable Opto
Controls	1.05 ± 0.50***	1.45 ± 0.71***	2.44 ± 1.23***			
UVH patients gain <0.20 early rehabilitation	2.24 ± 0.58	2.74 ± 0.79	3.67 ± 1.20	1.65 ± 0.55*	2.29 ± 0.77*	3.60 ± 1.40
UVH patients gain >0.20 early rehabilitation	2.64 ± 0.60	3.15 ± 1.16	3.87 ± 1.20	1.82 ± 0.57*	2.35 ± 0.81*	2.94 ± 1.19
UVH patients gain <0.20 late rehabilitation	2.61 ± 0.79	3.27 ± 0.80	4.53 ± 1.19	2.05 ± 0.66*	2.52 ± 0.78*	3.87 ± 1.21
UVH patients gain >0.20 late rehabilitation	2.54 ± 0.88	2.82 ± 0.80	4.13 ± 2.95	1.90 ± 0.71*	2.51 ± 0.69	3.52 ± 0.96

The table shows the mean (±SD) postural instability index evaluated with the wavelet analysis in the four sub-groups of patients with UVH (aVOR gain < or >020, early vs. late rehabilitation) tested on stable support with eyes open (EO), eyes closed (EC) and optokinetic stimulation (Opto), before and after rehabilitation with the rotatory chair protocol. Increased postural instability scores depending on the visual condition are observed, with significantly higher scores with conflicting visual cues (optokinetic) before and after rehabilitation as well. Improvement of the scores is seen in all sub-groups in EO and EC conditions. Data from age-matched healthy controls (N = 225) were significantly lower compared to the pre-rehabilitation scores of the patients with UVH. Significant differences with the controls or due to rehabilitation are indicated by asterisks. *p < 0.05; ***p < 0.001.

#### After Rehabilitation

As a rule, all postural parameters recorded in static posturography conditions were reduced after VR (*p* < 0.05). Table 2 (right side) provides the mean post-rehabilitation PII scores. A significant score decrease was found in the patients with UVH with (EO) or without (EC) vision, with PII values close to the healthy controls. By contrast, the patients still exhibited high values regarding all postural parameters recorded in the optokinetic condition (PII, SPD, CoP displacement amplitude, HF). Again, recovery of posture function depended neither on the degree of pre-rehabilitation vestibular hypofunction nor on the time delay between onset of symptoms and beginning of VR.

### Posture Control in Dynamic Posturography Conditions

#### Before Rehabilitation

**FIGURE 2:**
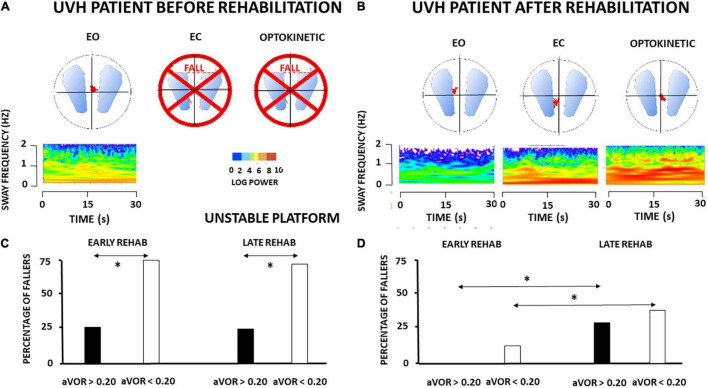
**(A–D)** Effect of vestibular rehabilitation (VR) on the postural performance of unilateral vestibular hypofunction patients in dynamic posturography conditions. Three-dimensional (3D) maps (wavelet analysis) of the antero-posterior stabilogram recorded in dynamic posturography on an unstable platform in one patient with UVH (early rehabilitation sub-group with aVOR gain above 0.20) examined before rehabilitation **(A)** and 4 weeks later, at the end of the last rehabilitation session **(B)**. Same legend as in Figure 1. The patient falls with eyes closed (EC) and optokinetic stimulation (Optokinetic) before rehabilitation. After rehabilitation near-normal balance function is recovered. The mean percentage of fallers when visual cues are excluded (EC) is illustrated for the four different subgroups of patients with UVH with early and late rehabilitation, aVOR gain below 0.20 (open histograms) and aVOR gain above 0.20 (filled histograms), before rehabilitation **(C)** and after rehabilitation **(D)**. Data showed significantly different percentages of fallers before rehabilitation as a function of the degree of vestibular hypofunction (higher in the sub-groups with aVOR gain <0.20), better dynamic balance recovery in the two sub-groups with early rehabilitation, and the best recovery profile (no fall) in the sub-group with both early rehabilitation and pre-rehabilitation aVOR gain >0.20. *p < 0.05.

In contrast with the static postural tasks, most of the patients with UVH examined in the most challenging conditions on unstable support remained unable to keep balance. They fell when vision was absent (EC) or altered (Optokinetic), in contrast to healthy controls who never fell in these visual contexts. Figure 2A shows the wavelet transform of the stabilograms of one representative patient with UVH on the unstable platform. Dynamic balance is possible with EO but falls are observed when vision is excluded or altered. Interestingly, significant differences were found depending on the degree of vestibular hypofunction on the weaker side. Figure 2C plots the histograms representing the percentage of fallers in the four sub-groups of UVH patients. A lower percentage was found in the two sub-groups (early and late rehabilitation) with aVOR gain above 0.20 (25 and 23%, respectively: filled histograms) compared to the two sub-groups (early and late) with aVOR gains below 0.20 (75 and 67%, respectively: open histograms; *p* < 0.001).

The ANOVAs performed on the different postural parameters provided by the non-linear analyses confirmed that dynamic postural performance depended on the degree of vestibular hypofunction. Significant differences were found between the sub-groups of patients with UVH with aVOR gains below 0.20 and the sub-groups with aVOR gains above 0.20 (*p* < 0.001).

**FIGURE 3:**
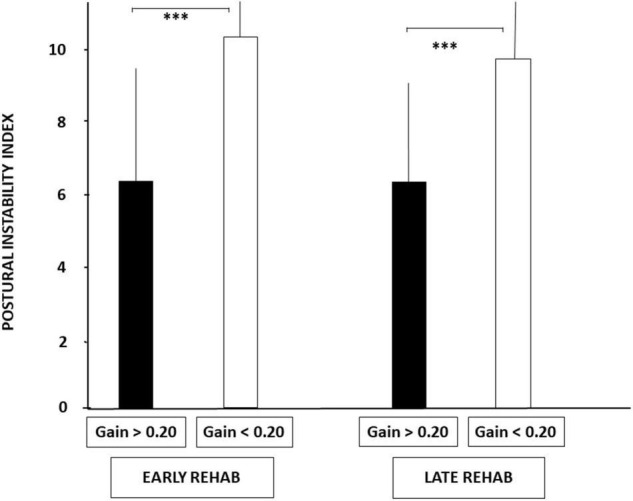
Increased postural instability before vestibular rehabilitation in UVH patients with a higher degree of vestibular hypofunction. The degree of vestibular loss on the weaker side was attested by the aVOR gain (see section “Materials and Methods”). Postural instability index (PII) derived from the wavelet analysis is illustrated for the stabilograms recorded in dynamic posturography (unstable platform) with eyes closed. Histograms show the mean (±SD) PII scores recorded in the early and late rehabilitation sub-groups with aVOR gain either above 0.20 (filled histograms) or below 0.20 (open histograms). Significant differences depending on the degree of vestibular hypofunction are shown by asterisks. ^***^p < 0.001.

Figure 3 illustrates the PII values recorded in the four sub-groups on unstable support with EC, a dynamic posturography condition highlighting the vestibular contribution to balance control. Lower PII scores were observed in patients with aVOR gains above 0.20. The mean PII scores calculated in the sub-groups with aVOR gain values above 0.20 were 6.38 ± 3.45 and 6.30 ± 3.0 for the early and late rehabilitation sub-groups, respectively, whereas they were significantly higher in the early and late sub-groups with aVOR gains below 0.20 (10.37 ± 2.97 and 9.80 ± 2.57, respectively; *p* < 0.001).

**FIGURE 4:**
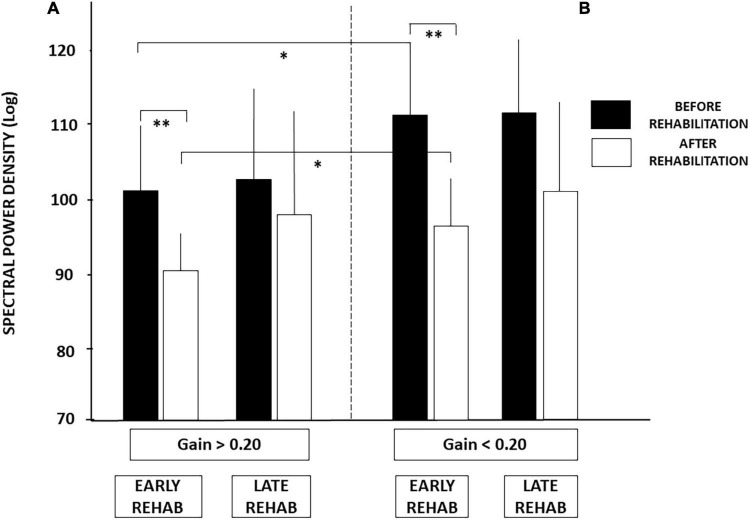
**(A,B)** Effect of both early rehabilitation and degree of pre-rehabilitation vestibular hypofunction on dynamic balance recovery in the patients with UVH; energy cost. Comparison of the recovery profiles in the four sub-groups of patients with UVH tested in dynamic posturography (unstable platform) with eyes closed. Mean spectral power density (SPD, ±SD, in decimal Log) calculated with the wavelet analysis is shown for both sub-groups with higher **(A)** and lower **(B)** pre-rehabilitation degree of vestibular hypofunction. SPD scores recorded before rehabilitation (filled histograms) and after rehabilitation (open histograms) are shown for both sub-groups with early and late vestibular rehabilitation. Better recovery depends on both early rehabilitation and the degree of vestibular hypofunction. Significant differences are indicated by asterisks. *p < 0.05; ^**^p < 0.01.

Similar conclusions can be drawn from the SPD values (cf. Figure 4), the period of posture stability without postural corrections calculated with the HF parameter, and the amplitude of the CoP displacement derived from the diffusion analysis (cf. Table 3). Patients with UVH with aVOR gains above 0.20 spent less energy for balance control, were stable over longer periods, and shifted to feedback control mechanisms for lower CoP displacements compared to patients with aVOR gain values below 0.20.

**TABLE 3:** Effect of vestibular rehabilitation on patients’ balance recovery in dynamic posturography condition.

	Hausdorff analysis (time, sec)		Diffusion analysis (CP amp, mm^2^)	
	Before rehabilitation	After rehabilitation	Before rehabilitation	After rehabilitation
UVH patients gain <0.20 early rehabilitation	3.96 ± 1.82*	2.18 ± 2.37	2282.4 ± 1155.7	547.8 ± 331.9
UVH patients gain >0.20 early rehabilitation	2.52 ± 1.54*	1.29 ± 0.80	1148.7 ± 681.1*	392.8 ± 281.0
UVH patients gain <0.20 late rehabilitation	3.77 ± 2.23*	2.21 ± 1.45	2203.1 ± 1302.4	887.5 ± 845.2
UVH patients gain >0.20 late rehabilitation	2.47 ± 0.76*	1.56 ± 0.40	1420.2 ± 772.8	836.5 ± 904.7

The table shows the mean data (±SD) evaluated with the fractal analysis and the diffusion analysis in the four sub-groups of patients with UVH (aVOR gain < or >0.20, early vs. late rehabilitation) tested on unstable support with eyes closed, before and after rehabilitation. The mean time-interval (seconds) during which patients are permanently unstable and continuously show balance corrections is provided by the fractal analysis. Significant differences are observed before rehabilitation as a function of the degree of vestibular hypofunction (lower scores in the sub-groups with aVOR gain >0.20 compared with sub-groups with aVOR gain <0.20). Significant improvement is seen after rehabilitation with the lowest score (best recovery) in the sub-group with early rehabilitation and aVOR gain above 0.20. The diffusion analysis looks less sensitive to discriminate the four sub-groups, very likely due to the great variability in the amplitude of CoP displacements (CP amplitude in mm^2^) for which patients shift from open-loop to closed-loop control mechanisms. *p < 0.05.

#### After Rehabilitation

Comparison of the 3D maps of one representative patient tested before and after rehabilitation shows that rehabilitation helps to restore balance function since the patient can perform the task in the most challenging conditions on unstable support with EC and moving visual cues (cf. Figures 2A,B). The percentage of patients with UVH who succeeded in these conditions was function, however, of the stage of rehabilitation and the degree of vestibular loss (cf. Figure 2D). The lower percentages of fallers were found in the sub-groups with early rehabilitation, and 100% of those with aVOR gain above 0.20 performed the postural tasks.

**FIGURE 5:**
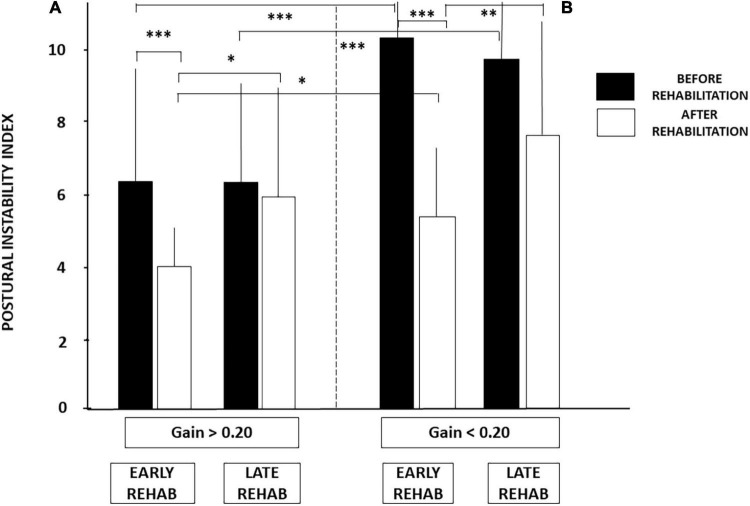
**(A,B)** Effect of both early rehabilitation and degree of pre-rehabilitation vestibular hypofunction on dynamic balance recovery in the patients with UVH; stability index. Comparison of the recovery profiles in the four sub-groups of patients with UVH tested in dynamic posturography (unstable platform) with eyes closed. Mean postural instability index (PII, ±SD) calculated with the wavelet analysis is shown for both sub-groups with pre-rehabilitation aVOR gain above **(A)** and below **(B)** 0.20. PII scores are shown before rehabilitation (filled histograms) and after rehabilitation (open histograms). Better recovery depends on both early rehabilitation and degree of vestibular hypofunction. Significant differences are indicated by asterisks. *p < 0.05; ^**^p < 0.01; ^***^p < 0.001.

Figure 5 illustrates how these two conditions (early vs. late rehabilitation, pre-rehabilitation aVOR gain below or above 0.20) impact dynamic balance recovery. The mean PII scores showed that balance recovery was better with early rehabilitation compared to late rehabilitation, whatever the pre-rehabilitation aVOR gain. For example, mean PII values decreased in the sub-groups with early rehabilitation from 6.38 ± 3.45 to 4.07 ± 1.04 (*p* < 0.009) when aVOR gain was above 0.20, and from 10.37 ± 2.97 to 5.18 ± 1.97 (*p* < 0.00001) when aVOR gain was below 0.20. No significant differences were found after rehabilitation in the two sub-groups with late VR (6.30 ± 3.0–5.95 ± 3.62 for the sub-group with higher aVOR gain, and 9.80 ± 2.57–7.54 ± 3.57 for the lower gain sub-group). The data also showed that dynamic balance recovery was better in the sub-group with early rehabilitation and higher aVOR gain (4.07 ± 1.04) compared with the sub-group with early rehabilitation and lower aVOR gain (5.18 ± 1.97; *p* < 0.03).

Analysis of the SPD scores led to similar conclusions (Figure 4). No significant differences were found after late rehabilitation while the lowest score attesting a better recovery was again found in the sub-group with both early rehabilitation and initial aVOR gain above 0.20 (101.04 ± 9.28 before rehabilitation compared to 91.45 ± 5.78 after rehabilitation; *p* < 0.04).

Results from the fractal and diffusion analyses are reported in Table 3. Great variability observed in the CoP amplitude parameter made the diffusion analysis less discriminating. On the other hand, the HF parameter confirmed that patients with pre-rehabilitation aVOR gain above 0.20 and early rehabilitation exhibited a shorter period of instability during the recording sessions (from 2.52 ± 1.54 s before rehabilitation to 1.29 ± 0.80 s after rehabilitation; *p* < 0.04) compared with significantly longer ones found in the subgroup with a pre-rehabilitation gain below 0.20 and late rehabilitation (from 3.77 ± 2.23 s to 2.21 ± 1.45 s; *p* < 0.05).

## Discussion

Taken together, this retrospective clinical study in patients with UVH extends to balance recovery the findings we recently highlighted for gaze stabilization recovery (Lacour et al., 2021). Balance function recovery also depends on the two conditions required to fully recover dynamic canal function, that is, early rehabilitation and degree of pre-rehabilitation vestibular hypofunction. This claim must, however, be restricted to dynamic postural tasks in which vestibular inputs predominate, that is, to challenging conditions encountered in daily life when visual or somatosensory cues are excluded or conflicting. In such contexts, the patients with early rehabilitation and pre-rehabilitation aVOR gain above 0.20 showed the best recovery profile.

### Balance Deficits and Recovery in Static Conditions

Posture control was strongly impaired in patients with unilateral vestibular loss compared with healthy controls, a finding that has long been known (Nashner et al., 1982; Black et al., 1983, 1988, 1989; Horak et al., 1990; Fetter et al., 1991; Curthoys and Halmagyi, 1995, 1999; Lacour et al., 1997; Horak, 2010). The advantage of non-linear analyses to investigate posture deficits and recovery has been fully explained in previous papers (Lacour et al., 2008, 2018). They provide more functional descriptors than the simple length and surface parameters used in clinical literature. They give access not only to precise quantification of posture stabilization (PII, wavelet transform), but they also seek to determine the energy spent to maintain quiet standing (SPD, wavelet transform), the feedback mechanisms controlling posture (CoP amplitude, diffusion analysis), and how often the body is stabilized over time (HF, fractal analysis). Our results showed that patients with UVH had significantly increased body sway, made postural corrections for larger body sway, spent more energy to maintain quiet standing, and were unstable during longer periods, compared to healthy controls. These are the reasons why the risk of falling is accentuated in the patients, even when tested in easy static postural tasks performed on a stable support.

Improvement of all postural parameters was observed after VR with the rotatory chair protocol, whatever the stage when rehabilitation was performed (early vs. late), thus confirming the prospective data collected on a smaller number of UVH patients (Lacour et al., 2020a). This absence of better recovery after early rehabilitation is in contrast with the critical period found for aVOR gain recovery (Lacour et al., 2019, 2020b,^2021^). It can be explained by the multisensory integration process necessary to achieve posture control in quiet standing conditions. In contrast to the aVOR, vestibular inputs are of less importance for posture control than more functional sensory cues from solar plant receptors, leg muscle proprioception, and vision (Lacour and Borel, 1993; Fitzpatrick and McCloskey, 1994; Mergner and Rosemeier, 1998; Curthoys et al., 2017). Visual and somatosensory substitution mechanisms have been reported many times in the vestibular compensation literature, and the powerful reweighting of visual cues was particularly highlighted (Nashner et al., 1982; Fetter et al., 1991; Horak and Shupert, 1994; Lacour et al., 1997; Curthoys, 2000). The poor recovery observed when visual cues are conflicting (optokinetic stimulation) also supports the general claim that visual substitution is a strong sensory substitution mechanism (Dichgans et al., 1976; Paulus et al., 1984; Diener et al., 1986; Bronstein, 1995; Young et al., 2012), so strong that it may lead sometimes to visual dependency (Bronstein, 1995). The strength of the sensory substitution processes can explain why the degree of vestibular hypofunction and stage of rehabilitation is not crucial to compensate for postural deficits in easy postural conditions. Daily life activity after vestibular loss provides natural conditions to use the sensory feedback required to compensate. The advantage of early rehabilitation remains, however, and can speed up the natural compensation process (Whitney et al., 2015) and correct maladaptive postural strategies reported by Horak et al. (1990).

### Balance Deficits and Recovery in Dynamic Conditions

In most challenging conditions on unstable support with altered visual cues (EC, Optokinetic), the patients with UVH before rehabilitation have difficulties performing the task and can fall. The percentage of fallers was lower in the sub-groups with pre-rehabilitation aVOR gain above 0.20 (around 25% with EC) compared with those with aVOR gain below 0.20 (around 70–75% with EC) (*p* < 0.001; cf. Figure 2). After early rehabilitation, all patients with higher aVOR gain could perform the postural task while the mean percentages of fallers remained significantly higher for the sub-groups with late rehabilitation (28 and 35% for the sub-groups with high and low aVOR gains, respectively). This behavioral observation and the postural parameters derived from the non-linear analyses indicate that both stages of rehabilitation and degree of vestibular hypofunction matter for dynamic balance recovery.

The differences observed in both impairment and recovery of dynamic balance function between patients with high and low pre-rehabilitation aVOR gains is a new finding. Balance deficits were less severe and balance recovery was better in patients with pre-rehabilitation aVOR gain above 0.20 compared with those with gain below 0.20. All postural parameters derived from the non-linear analyses confirmed this statement. The predominant role of vestibular input in dynamic postural tasks could be one explanation: functional vestibular asymmetry is lower with weaker side gain above 0.20, and the dynamic balance deficits less severe, compared with higher asymmetry with hypofunction side gain below 0.20, and more pronounced balance deficits. As reported for dynamic canal function recovery, early rehabilitation is a necessary but not sufficient condition. Degree of vestibular hypofunction matters too (Lacour et al., 2021).

The beneficial effect of early sensorimotor activity on behavioral compensation has been fully discussed in our animal models (Lacour et al., 1976; Xerri and Lacour, 1980). Briefly, training and sensory-motor activity reinforce and optimize vestibular lesion-induced neural plasticity if done during a post-lesion sensitive period (first 2 weeks after symptoms onset) during which plasticity mechanisms were re-expressed (Lacour and Tighilet, 2010; Lacour et al., 2015). Plastic events are tuned dynamically according to post-lesion experience and training. Hebbian neural plasticity varies across post-lesion time and depends on the quantity of remaining afferent terminals (Gall and Lynch, 1981; Allred et al., 2014). The time constant of both axonal sprouting-induced new terminals and postsynaptic receptors proliferation coincides with the post-lesion critical period for VR. Vestibular lesion-induced synaptic remodeling and neural repair have been described at the peripheral level [(Gaboyard-Niay et al., 2016): sensory epithelium] and more centrally [(Lacour and Tighilet, 2010): vestibular nuclei], suggesting that synaptic reorganization should be better in patients with UVH with pre-rehabilitation gain above 0.20 and early rehabilitation. Indeed, as discussed for recovery of gaze stabilization (Lacour et al., 2021), aVOR gain can be fully restored if remaining semicircular canal afferents are in a sufficient number. Canal and otolith inputs converge onto vestibular nuclei neurons projecting to the spinal cord *via* vestibulo-spinal pathways implicated in dynamic balance function (Black and Nashner, 1984; Allum et al., 1988). Reweighting of canal input on the hypofunction side could explain the better balance recovery with early rehabilitation and pre-rehabilitation aVOR gain above 0.20.

These findings strongly suggest two clinical recommendations: (1) patients should be referred to ears, nose, and throat (ENT) specialists as soon as possible after vertigo attack, and (2) ENTs should refer patients to a physiotherapist with proper expertise in VR as quickly as possible.

### Limits of the Study

Limitations of the study mainly concern the otolith contribution to posture and balance function, not investigated here. Utricle and saccule status remains unknown in our patients with UVH. Further analyses incorporating vestibular evoked myogenic potentials are needed to investigate the hypothesis that better dynamic balance recovery could also result from otolith reweighting on the weaker side. Another limitation is the small size of the different sub-groups; a study on a wider sample of patients remains to be done, and the role of both gender of patients and side of the vestibular pathology should be also investigated.

## Data Availability Statement

The raw data supporting the conclusions of this article will be made available by the authors, without undue reservation.

## Ethics Statement

The studies involving human participants were reviewed and approved by the CCPPRB Nice. The patients/participants provided their written informed consent to participate in this study.

## Author Contributions

All authors listed have made a substantial, direct, and intellectual contribution to the work, and approved it for publication.

## Conflict of Interest

The authors declare that the research was conducted in the absence of any commercial or financial relationships that could be construed as a potential conflict of interest.

## Publisher’s Note

All claims expressed in this article are solely those of the authors and do not necessarily represent those of their affiliated organizations, or those of the publisher, the editors and the reviewers. Any product that may be evaluated in this article, or claim that may be made by its manufacturer, is not guaranteed or endorsed by the publisher.
